# Complications and risk factors in transrectal ultrasound-guided prostate biopsies

**DOI:** 10.1590/S1516-31802006000400005

**Published:** 2006-05-04

**Authors:** Carlos Márcio Nóbrega de Jesus, Luiz Antônio Corrêa, Carlos Roberto Padovani

**Keywords:** Needle biopsy, Prostatic neoplasms, Risk factors, Ultrasonography, Prostate, Biópsia por agulha, Câncer de próstata, Fatores de risco, Ultrasonografia, Próstata

## Abstract

**CONTEXT AND OBJECTIVE:**

Prostate biopsy is not a procedure without risk. There is concern about major complications and which antibiotics are best for routine use before these biopsies. The objective was to determine the rate of complications and the possible risk factors in prostate biopsies.

**DESIGN AND SETTING:**

Prospective study, Facul-dade de Medicina de Botucatu.

**METHODS:**

Transrectal ultrasound (TRUS) guided prostate biopsies were carried out in 174 patients presenting either abnormality in digital rectal examinations (DRE) or levels higher than 4 ng/ml in prostate-specific antigen (PSA) tests, or both.

**RESULTS:**

Hemorrhagic complications were the most common (75.3%), while infectious complications occurred in 19% of the cases. Hematuria was the most frequent type (56%). Urinary tract infection (UTI) occurred in 16 patients (9.2%). Sepsis was observed in three patients (1.7%). The presence of an indwelling catheter was a risk factor for infectious complications (p < 0.05). Higher numbers of biopsies correlated with hematuria, rectal bleeding and infectious complications (p < 0.05). The other conditions investigated did not correlate with post-biopsy complications.

**CONCLUSIONS:**

Post-biopsy complications were mostly self-limiting. The rate of major complications was low, thus showing that TRUS guided prostate biopsy was safe and effective. Higher numbers of fragments taken in biopsies correlated with hematuria, rectal bleeding and infectious complications. An indwelling catheter represented a risk factor for infectious complications. The use of aspirin was not an absolute contraindication for TRUS.

## RESUMO

**CONTEXTO E OBJETIVO:**

A biópsia da próstata não é um procedimento isento de riscos. Existe preocupação com respeito às complicações e quais seriam os melhores antibióticos usados antes do procedimento. O objetivo foi determinar a taxa de complicações e os possíveis fatores de risco para complicação na biópsia da próstata.

**TIPO DE ESTUDO E LOCAL:**

Estudo prospectivo clínico, realizado no Hospital das Clínicas de Botucatu.

**MÉTODOS:**

Foram realizadas biópsias em 174 pacientes que apresentavam anormalidade ao exame digital da próstata ou antígeno prostático específico maior que 4 ng/ml ou ambos. Todos os pacientes realizaram enema e antibioticoprofilaxia previamente ao exame. As complicações foram anotadas após o término do procedimento e em consultas posteriores. Algumas condições foram investigadas como possíveis fatores de risco para biópsias de próstata: idade, câncer da próstata, diabetes melito, hipertensão arterial sistêmica, antecedentes de prostatite, uso de ácido acetilsalicílico, volume prostático, número de biópsias e uso de sonda vesical.

**RESULTADOS:**

As complicações hemorrágicas foram mais comuns (75,3%) enquanto que as infecciosas ocorreram em 19% dos casos. O tipo mais freqüente foi a hematúria, ocorrendo em 56% dos pacientes. A infecção do trato urinário ocorreu em 16 pacientes (9,2%). Sepse foi observada em três pacientes (1,7%). Não houve óbitos. Em 20% dos pacientes não foram observadas complicações após o exame. A presença da sonda vesical foi fator de risco para complicações infecciosas (p < 0,05). O número maior de amostras nas biópsias foi relacionado à hematúria, sangramento retal e complicações infecciosas (p < 0,05). As demais condições investigadas não se relacionaram com complicações pós-biópsia da próstata.

**CONCLUSÕES:**

As complicações pós-biópsia da próstata foram em sua maioria autolimitadas. A taxa de complicações graves foi baixa, sendo a biópsia de próstata guiada pelo ultra-som segura e eficaz. A retirada de um maior número de fragmentos na biópsia relaciona-se com hematúria, sangramento retal e complicações infecciosas. A sonda vesical foi um fator de risco para complicações infecciosas.

## INTRODUCTION

Prostate cancer diagnoses have noticeably increased recently. It was estimated that 234,460 new cases were diagnosed in the USA in 2006.^1^ Consequently, there is growing interest in prostate cancer diagnosis, treatment and control, making it a public health concern.

The main tests used for prostate cancer detection, such as digital rectum examination (DRE) and prostate-specific antigen (PSA), present flawed diagnoses, particularly among patients who have PSA of between 4 and 10 ng/ml, with a false negative rate of 20 to 30%.^2,3^ Therefore, the method of choice for obtaining a conclusive diagnosis is transrectal ultrasound (TRUS) guided prostate biopsy.^2–4^ However, the possibility that prostate cancer is present is not totally excluded even with a negative result.

Although TRUS presents low rates of complications and good tolerance,^5,6^ it is an invasive procedure that is not free from inherent complications, and it is often difficult and painful for the patients.^7,8^ For all these reasons, there is great interest in making the procedure fast, safe and effective, with a low rate of complications.

## OBJECTIVE

Our aim was to assess locally the main complications, possible risk factors, outcomes, safety and effectiveness of TRUS-guided biopsies.

## PATIENTS AND METHODS

This was a prospective study involving 177 patients who spontaneously sought our urology service from March 2000 to June 2001. During the medical consultation, we registered personal data, clinical history, DRE and PSA values, and possible risk factors for post-biopsy complications, such as urinary tract infection (UTI), systemic arterial hypertension, diabetes, indwelling catheter, use of acetylsalicylic acid and prostatitis antecedents.

The criteria for biopsy indication were abnormality in DRE and/or high PSA values, considering 4 ng/ml to be the maximum limit for normality (Tandem^®^, Hybritech Inc). In cases with signs or symptoms of possible UTI, or a previous positive urine culture, a new urine culture was requested following the appropriate treatment. Such patients were only biopsied after negative urine culture results had been obtained.

The first 106 patients were administered sulfamethoxazole (800 mg) and trimethoprim (160 mg) twice a day, beginning two days before the procedure and continuing for five days afterwards, thus totaling seven days of prophylaxis. The last 71 patients were prescribed short-duration prophylaxis consisting of 500 mg of ciprofloxacin two hours before and eight hours after the procedure. Before the biopsies were taken, all patients underwent rectal clyster.

The biopsies were performed on an outpatient basis by two urologists (C.M.N.J. and L.A.C.). The procedure was guided by Dornier ultrasound equipment, with a 6.5 MHz multiplanar transrectal probe using the Biopty Gun^®^. Before the biopsies, DRE was performed once more, in an attempt to associate any abnormality with possible alterations in the TRUS image observed. The volume of the prostate was calculated by means of the revolution ellipsoid formula (height x width x length x 0.52) in cubic centimeters.^9^ Sampling was done per sextant, with additional sampling in suspicious areas such as hypoechogenic areas or those with loss of capsular limits.^10^ In prostates larger than 60 cm^3^ or in cases of repeated biopsy, the number of samples taken was increased. No sedation was applied, or any local periprostatic anesthesia; only lido-caine gel at the moment when the probe was introduced. The biopsied fragments were sent separately for histopathological examination, in vials containing formalin.

After one week, all the patients were assessed in accordance with the study protocol, regarding their symptomatology and post-biopsy complications. After one month, the patients were notified about the histopathological results and respective medical decision.

For the statistical analyses of the variables, Goodman's test for associations between two classified qualitative variables was utilized, and also two non-parametric tests: the Mann-Whitney test for two independent groups and Kruskal-Wallis test for more than two groups of independent variables. All analyses were done using a sig nificance level of 5%.

## RESULTS

The mean age of these 177 patients was 68.2 ± 8.1 years (range: 47-87). Among them, three were excluded: two patients because of irregular use of prophylactic antibiotic and another one because of concomitant use of another antibiotic due to tonsillitis contracted after the prostate biopsy was taken.

Serum PSA values were determined prior to the biopsies in 158 patients (90.8%), and their mean value was 18.5 ng/ml ± 38.7 (range: 0.2-302 ng/ml). The mean volume of the prostate was 47 cm^3^ ± 31.2 (range: 8-325 cm^3^). The mean number of biopsies was 7.5 ± 2.6 (range: 3-17). In patients with prostates with less than 60 grams (n = 135), the mean number of samples per procedure was 7.2 ± 2.3. In those with prostates larger than 60 grams (n = 39), it was 8.8 ± 3. Prostate cancer was diagnosed in 43 patients (23.7%).

Table 1 shows the frequencies of possible risk factors for complications in TRUS guided biopsy. Only 36 patients did not present any complication after prostate biopsy (20.7%). It was found that hemorrhagic complications were the most frequent event in 131 patients (75.3%), while only 25 cases presented infectious complications (14.4%) (Table 2). Prostate volume, age, use of acetylsalicylic acid, arterial hypertension, previous history of prostatitis, diabetes mellitus and presence of CAP did not increase the probability of any post-biopsy complication.

**Table 1.** Risk factors for complications in transrectal ultrasound-guided prostate biopsy in 174 patients

Risk factors	Number of patients	(%)
**Diabetes mellitus**	26	15
**Arterial hypertension**	61	35.1
**Bladder catheter**	23	13.2
**Use of acetylsalicylic acid**	16	9.2
**History of prostatitis**	25	14.4
**Prostate cancer**	43	23.7

**Table 2.** Complications following transrectal ultrasound-guided prostate biopsy in 174

Complications	Number	(%)
**Hemorrhagic complications**	131	75.3
hematuria	98	56.3
rectal bleeding	57	32.3
hematochezia	25	14.4
hemospermia	38	21.8
urethral bleeding	1	0.6
**Infectious complications**	25	14.4
dysuria	11	6.3
frequency	7	4.0
fever	11	6.3
shivers	5	2.9
positive urine culture	16	9.2
asymptomatic bacteriuria	6	3.4
orchitis	1	0.6
prostatitis	1	0.6
**Major complications**	5	2.9
Sepsis	3	1.7
gross hematuria	1	0.6
urine retention	1	0.6
Hospitalization	4	2.3
**No complication**	36	20.6

Hematuria was the most frequent sign in all complications, corresponding to 56.3% of the cases. Among the hemorrhagic events, rectal bleeding corresponded to 32.8% (57 cases) and hemospermia to 21.8% (38 cases). Urethral bleeding was rare, observed in only one case (0.4%). Periprostatic and perineal hematomas were not observed (Table 2).

The rate of major complications was low (1.8%). Among the major complications, one was hemorrhagic, three were infectious with sepsis and one was urine retention. Except for this latter case, all these patients needed medical intervention with hospitalization (2.3% of the total number of patients). The length of hospitalization ranged from two to seven days, with no mortality.

Among the major and most frequent complications, hematuria was a self-limiting component. Only one case out of 98 patients needed medical intervention and hospitalization (1%). A large majority (74.4%) presented slight hematuria that ceased after the third day. Only 5.1% presented hematuria for more than one week.

Rectal bleeding was the second most frequent hemorrhagic complication, occurring in 92% of the cases during the first two days after the procedure. It was more associated with non-diabetic patients (36.5%) than with diabetic patients (11.5%) (p < 0.05).

Another interesting finding was the incidence of hemospermia. Although only 21.8% reported it (38 patients), its incidence rose almost fourfold when considering only the patients who reported sexual intercourse during the first month after the biopsy.

It was statistically significant that the average number of samples was higher in patients with hematuria and rectal bleeding than in those who did not present these signs (p = 0.05 and p = 0.02, respectively). This was not found in patients with hemospermia, or in the overall data for all hemorrhagic complications (p = 0.3 and p = 0.61, respectively).

Infectious complications affected 25 patients (14.4%). UTI was the most common one (9.2%), although it was asymptomatic in 37.5% of the cases. *Escherichia coli* was the most common bacterium in eight patients. There were correlations between infectious complications and the use of indwelling catheters and high numbers of samples (p < 0.05) There was also positive correlation of high numbers of samples with feverish patients (p < 0.05). The other factors studied did not influence the incidence of this kind of complication (Table 3).

**Table 3.** Results from statistical tests relating to possible risk factors for complications following transrectal ultrasound-guided prostate biopsy

Possible risk factor	Infectious complications	Hemorrhagic complications
Age (more than 65 years old)	-	-
Diabetes mellitus	-	-
Arterial hypertension	-	-
Previous prostatitis	-	-
Prostate cancer	-	-
Number of samples	+	+*
Prostate volume	-	-
Use of acetylsalicylic acid	-	-
Bladder catheter	+	-

*(-) negative sign: not significant; (+) positive sign: significant with p < 0.05.*

* *Hematuria and rectal bleeding with p = 0.05 and p = 0.02, respectively.*

## DISCUSSION

Although TRUS-guided prostate biopsy is currently regarded as the ideal method for obtaining prostate fragments for histological analysis, it is considered to be an invasive procedure that is rather uncomfortable for patients.^6–8^ Since most men undergoing biopsy are fit and economically active, there is major interest in making the procedure as safe and effective as possible, thus minimizing complications whenever possible.

Complications also mean higher costs. Kapoor et al.^11^ demonstrated that prophylaxis with ciprofloxacin can reduce post-biopsy costs by 26 dollars per patient, when compared to placebo.

The overall rate of post-biopsy complications was 79.3%. Among these, only 2.9% were considered major complications, thus attesting that TRUS-guided prostate biopsy is a safe procedure. Most of the minor complications were self-limiting and disappeared a few days after the biopsy, without producing greater morbidity. Such findings have also been reported by other authors like Rodriguez and Terris,^6^ who found at least one complication in 63.6% of the patients involved in their clinical trial. The importance of our finding is that it indicates that four out of five biopsied men may have some kind of post-biopsy complication. Patients should have access to this information before undergoing the procedure.

Among the most common minor complications, hematuria has been found to be the most frequent one, in 12 to 80% of biopsied patients.^5,6,8,12-15^ Our data showed that the incidence of hematuria was 56.3% (98 out of 174 patients). These figures are compatible with the literature, and particularly with studies that were conducted prospectively, with active assessment, either via questionnaires or via interviews (Table 4).

**Table 4.** Complications and risk factors following transrectal ultrasound, as reported in the literature and in the present study

Authors	year	Patient sample size	Hematuria (%)	Rectal bleeding (%)	Hemospermia (%)	Fever (%)	Urinary tract infection (%)	Sepsis (%)	Hospitalization (days)
Clements et al.^5^	1993	80	20.0	9.4	5.1	2.5	-	-	0.004
Aus et al.^12^	1996	391	13.0	-		3.9	7.5	-	-
Rietbergen et al.^15^	1997	1,687	49.6	2.8	45.3	4.2	7.3	0.2	0.4
Rodriguez and Terris^6^	1998	127	57.0	10.0	9.5	1.7	2.5	0	-
Deliveliotis et al.^18^	1999	120	65.0	33.3	29.1	6.6	8.1	0.16	0.16
Raaijmakers et al.^14^	2002	5,676	22.6	1.3	50.4	3.5	6.9	0.004	0.5
Present series	2004	174	56.3	32.3	21.8	6.3	9.2	1.7	2.3

Few cases of hematuria have been reported in retrospective studies in which the focus was not the post-biopsy complications.^16^ Some studies have only considered that cases presented hematuria when it lasted for more than three days.^14,17^ According to our findings, such situations constituted only one-fourth of the cases.

Rectal bleeding was the second greatest cause of hemorrhagic complications in our study, corresponding to 32.8% of the patients undergoing biopsy (57 out of 174). Other studies have reported incidence ranging from 1.3% to 37.1% of the biopsied patients.^5,6,8,12,14,15^ The big difference between the results found in the literature and our results lies in the concept of rectal bleeding, the duration of the bleeding and the patient's difficulty in noticing this sign. Conceptually, rectal bleeding originates from the rectal ampulla and is characterized by the presence of live blood from the anus. In our study, rectal bleeding was taken to mean any kind of bleeding from the anus, either observed in spontaneous release from the anus or blood stains on the underwear, or even by observation of blood in the feces (hematochezia). In some publications, the only sign considered is hematochezia, which has lower incidence than the rectal bleeding itself.^6,13,17^ Our sample of patients presented 25 cases of hematochezia (14.4%), which corresponded to less than half of the cases of rectal bleeding.

Few reports in the literature deal with the duration of post-biopsy rectal bleeding. Deliveliotes et al.^18^ reported only that hematochezia did not last for more than six days. In our study, 92% of the cases of rectal bleeding were observed within the first two days following the procedure, but never for longer than seven days.

In the literature, there is no standardization in the descriptions of infectious complications. Furthermore, depending on the methodology utilized, the rate of infectious complications may seem to increase or decrease. However, infectious complications are less frequent than hemorrhagic ones, although they present higher potential morbidity.

Twenty-five patients (14.4%) presented at least one type of infectious complication. Post-biopsy UTI was the most frequent single complication among the infectious complications, and was found in 9.2% of the men (16 out of 174). Six out of these 16 cases of urinary infection (37.5%) were totally asymptomatic. Asymptomatic bacteriuria was described by Fong et al.^19^ in 7% of their patients when two types of antibiotics were compared. Ruebush et al.^20^ demonstrated that asymptomatic bacteriuria only occurred in seven patients using placebo (10.7%), whereas there were no occurrences in the group that used sulfa-methoxazole-trimethoprim. The presence of a positive urine culture is associated with antibiotic prophylaxis, type of antibiotic utilized and presence of UTI before the biopsy.^14,21,22^

*Escherichia coli* was the most common bacterium, responsible for 50% of the positive urine cultures (8 out of 16). There were no infections caused by anaerobic germs and only one infection caused by Gram-positive germs (coagulase-positive *Staphylococcus*). If the frequency of hospitalization only among patients with positive urine cultures is considered, it can be seen that its probability is 13 times higher than the hospitalization rate among all the patients involved in our study. Kapoor et al.^11^ reported a similar relationship through observing that one in four of their patients needed hospitalization in cases of asymptomatic bacteriuria, thus demonstrating that this complication must be strongly avoided.

Interruption of the use of thrombolytic agents and medications affecting platelet function is a common practice among surgeons in general and among those who perform prostate biopsies. Such drug suspensions, lasting for seven to ten days, are usually implemented even for patients who need to take these medications, such as coronary patients and those who have suffered ischemic strokes. Since great numbers of patients in these conditions have to undergo prostate biopsies, there is a concern that the suspension of the drug may bring about problems with regard to prevention of thromboembolic phenomena. In one study, the use of acetylsalicylic acid did not increase the incidence of any type of hemorrhagic complications, in spite of the small number of patients.^16^ Similar data were reported by Rodriguez and Terris.^6^

The number of samples taken is also a controversial issue as a risk factor in prostate biopsies. In some studies in the literature, the additional number of biopsies could not be associated with any increase in the numbers of hemorrhagic complications. Naughton et al. compared the morbidity caused by collecting six and twelve fragments in prostate biopsies and found no significant difference.^13^ An additional seventh biopsy did not produce any increase in morbidity according to Raaijmakers et al.^14^

In our study, in spite of not finding any relationship between the number of samples and the total of number of hemorrhagic and hemospermia complications, there was an association between the numbers of samples with hematuria and rectal bleeding. The explanation for this fact is that, with a higher number of samples, the probability of injuring a prostatic and/or rectal blood vessel is higher, thereby causing hematuria and/or rectal bleeding. Higher numbers of samples were not related to hemospermia, which can be explained by the fact that most patients did not report ejaculations following the biopsy. Otherwise, the results might have been similar to those from patients with hematuria and rectal bleeding.

Increased numbers of samples were also correlated with increased rates of infectious complications. The number of samples was a facilitative factor for infections of the urinary tract. One explanation for this is the increasing probability, with each new sampling action, of contamination from the rectal ampulla into the prostate. This contamination would be proportionally smaller when the number of samples was less than or equal to six fragments. The action of multiple sampling may also facilitate the penetration of microorganisms into the bloodstream from a prostate with latent germs, thereby favoring an infectious process. To reinforce this hypothesis, we cite the cases of three patients who developed sepsis and needed hospitalization. These patients had undergone 12, 13 and 17 prostate fragment-sampling actions.

Furthermore, when the higher mean number of samples from feverish patients (9.2 ± 3.5) was compared with the lower number from non-feverish patients (6.1 ± 1), this difference was statistically significant. Although the number of samples that leads to an increased possibility of infections is not known, an antibiotic with a wide spectrum of action, good prostatic penetration and high urine and serum concentrations should be used when a high number of samples are needed.

Finally, the presence of a bladder catheter (urethral catheter or cystostomy) is an additional risk factor for the occurrence of complications in patients undergoing prostate biopsies. It is believed that the presence of the catheter, as a foreign body in the urinary tract, favors the proliferation of pathogenic microorganisms, even if the urine cultures are negative. Among 23 of our patients with catheters, nine presented infectious complications (39.1%). Aus et al.^12^ also observed that patients with bladder catheters had 2.3 times more risks of urinary infection than did patients without that risk factor. In our sample of patients, this risk was 3.6 times higher.

## CONCLUSIONS

Post-prostate biopsy complications were mostly self-limiting and did not require medical intervention. The rate of major complications was low, and hence transrectal ultrasound (TRUS) guided prostate biopsy is a safe and effective procedure. However, high numbers of biopsied fragments were correlated with hematuria, rectal bleeding and infectious complications. The use of bladder catheters was also a risk factor for infectious complications. The use of aspirin was not a contraindication for TRUS.
